# Efficient synthesis of 5-substituted 2-aryl-6-cyanoindolizines via nucleophilic substitution reactions

**DOI:** 10.1186/1860-5397-1-9

**Published:** 2005-10-07

**Authors:** Eugene V Babaev, Natalya I Vasilevich, Anna S Ivushkina

**Affiliations:** 1 Department of Chemistry, Moscow State University, 119992, Moscow, Russia

**Keywords:** 5-chloroindolizine, 5-substituted indolizines, 5-indolizinone, nucleophilic substitution

## Abstract

2-Aryl-6-cyano-7-methyl-5-indolizinones were successfully converted into 2-aryl-5-chloro-6-cyano-7-methylindolizines. The obtained 5-chloroindolizines readily underwent nucleophilic substitution at position 5 leading in high yields to novel 5-functionalised indolizines.

Indolizines are an important class of heterocyclic compounds since many natural alkaloids contain in their structure a saturated (swainsonine) or aromatic (camptothecin) indolizine moiety. While the chemistry of indolizines has been widely investigated[1] the chemistry of 5-substituted indolizines remains very poor because there are only a few reliable ways for their synthesis.

8-Nitroindolizines may undergo amination at position 5 (S_N_H substitution) under the action of secondary amines.[2] 2-Phenylindolizine can be lithiated at position 5, and the resulting indolizyl lithium can react with some electrophiles (CO_2_, PhCHO, PhCN, Me_3_SiCl, MeI) leading to variety of new products in good yields.[3] An interesting method for preparing 5-substitutied indolizines by recyclization of oxazolo[3,2-a]pyridinium salts was developed in our laboratory.[4–5] Using this strategy a series of 5-substituted indolizines have been prepared in good yields, but (although the method seems to be quite reliable) it is currently restricted only by secondary amines.

In seeking for a synthetic approach to 5-substituted indolizines we have assumed that indolizines bearing an appropriate leaving group (*e.g.* halogen) at position 5 may undergo nucleophilic substitution. Herein we discuss the synthesis of previously unknown 5-chloroindolizines and their use as precursors to novel 5-substituted indolizines via nucleophilic displacement reactions.

The synthesis of 2-aryl-5-chloro-6-cyano-7-methylindolizines **2** is shown in Scheme 1. 2-Aryl-6-cyano-7-methyl-5-indolizinones **1 a** – **d** were prepared according to protocol of Gevald.[6] Our modification of the original method included separation of N- and O-isomers of phenacyl pyridines before cyclization (using the difference in their solubility in chloroform). Although ^1^H-NMR (see Supporting Information File 1) and Nuclear Overhauser Effect confirmed the structure **A** for indolizinones **1**, we assumed the existence of tautomerism between forms **A** and **B** involving hydrogen interchange between oxygen and C-3 carbon (Scheme 1). Although the amount of tautomer **B** is negligibly small, one would expect that treatment of **1 a** – **d** with phosphorous oxychloride may lead to substitution of oxo/oxy-group to chlorine giving the products **2 a** – **d**. (It is well known that analogous 2-hydroxypyridines which exist in the pyridone tautomeric form can be easily converted to 2-chloroderivatives by reaction with POCl_3_.).[7] Indeed, heating of indolizinones **1 a** – **d** in POCl_3_ at 80–100°C during 10 hours without any solvent followed by pouring into a mixture of ice/sodium acetate and filtration of the green precipitate afforded crude 5-chloroindolizines. After column chromatography (eluent – carbon tetrachloride) yellow solids were obtained. Performing this reaction in the presence of two-fold molar excess of trimethylbenzylammonium chloride or TEBAC increased the yields of **2 a** – **d** up to 30–75%. In the ^1^H NMR spectra of these products^†^ the initial signal of 3-CH_2_ group at ~5 ppm (intensity 2H) disappeared, and a new aromatic signal 3-CH (with intensity 1H) appeared at 7.99 – 8.11 ppm.

**Scheme 1:**
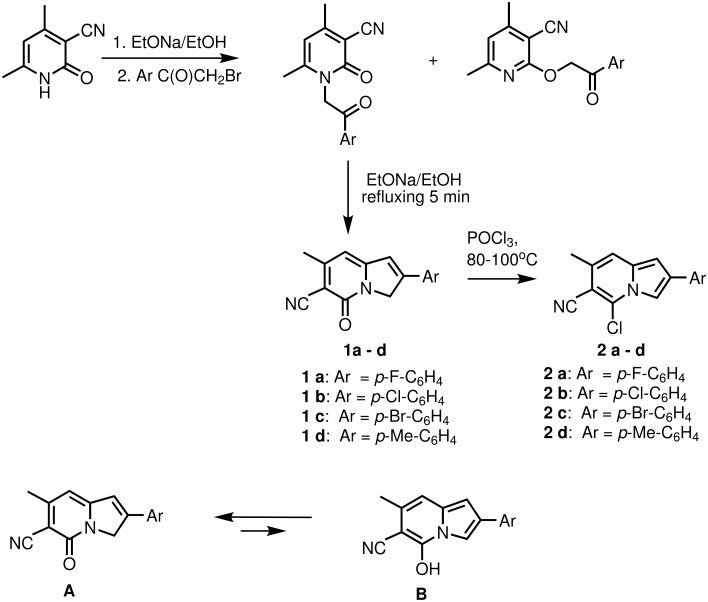
Synthesis of 2-aryl-5-chloro-6-cyano-7-methylindolizines **2**. Possible tautomeric structures **A** and **B** for 2-aryl-6-cyano-7-methyl-5-indolizinones **1**.

The halogen atom in 5-chloro-6-cyanoindolizines **2** should be activated to nucleophilic substitution reactions by the suitable *ortho*-arrangement of the nitrogen atom of the pyridine ring and electron-withdrawing cyano-group. The pattern strongly resembled 2-chloro-3-cyanopyridine, that is why we anticipated successful substitution in reactions of **2** with oxygen, nitrogen, and sulfur nucleophiles. Indeed, 5-chloroindolizines readily underwent nucleophilic substitution to produce previously unknown compounds **3** – **6** in good to excellent yields (Scheme 2). These products are detailed in Table 2. Thus, 5-methoxyindolizines **3 a** – **c** were formed after refluxing **2 b** – **d** in solution of sodium methoxide in methanol overnight in good yields (Scheme 2). Treatment of **2a**, **d** with excess of amines without any solvent gave 5-amino derivatives **4 a** – **h**. In the case of secondary amines (**4 a** – **c**, **e** – **g**) the reaction proceeded at room temperature, but reaction with less nucleophilic benzylamine (**4 d**, **h**) required heating for 30 min. Nucleophilic substitution also occured with sulfur nucleophiles. Thus, **2d** reacted with mercaptoethanol under basic conditions leading to **5a**. Conversion **2a** into **5b** was conveniently achieved with ethyl mercaptoacetate in ethanolic sodium hydroxide. Interestingly, **2a** reacted also with thiourea in refluxing butanol giving indolizinethione **6**. The product **6** seems to be the result of decomposition of unstable isothiouronium salt, and the process resembles the known conversion of 2-chloro-3-cyanopyridines to 3-cyanopyridinethiones under the same conditions via a similar intermediate.[8]^1^H NMR spectrum of **6**, which was very similar to the spectra of **1**, indicated disappearance of aromatic proton signal H_3_ and appearance of a signal at 5.35 ppm with intensity 2H.^†^

**Scheme 2:**
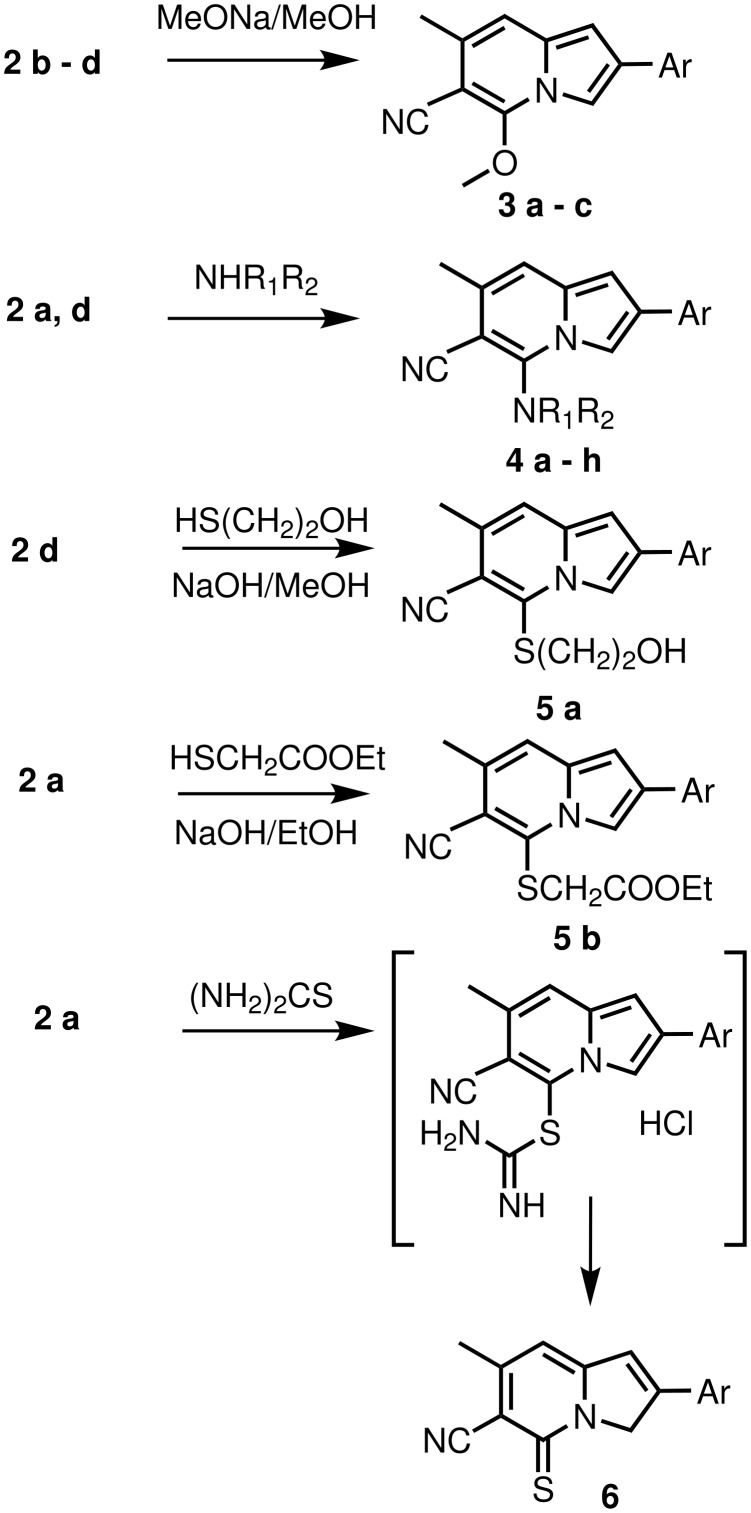
Nucleophilic substitution in 2-aryl-5-chloro-6-cyano-7-methylindolizines.

In conclusion, we are the first to obtain 2-aryl-5-chloro-6-cyano-7-methylindolizines from 2-aryl-6-cyano-7-methyl-5-indolizinones and to prove the possibility to employ them in nucleophilic substitution reactions. Moreover, these reactions are the first examples of preparative nucleophilic substitution in indolizines, and our findings open a new way to functionalize the C-5 position (in most cases considered as inactive). The studies of further cyclizations of 5-substituted indolizines involving neighbouring cyano-group and ring position C_3_ is underway.

**Table 1:** Properties of 5-substituted 2-aryl-6-cyano-7-methylindolizines

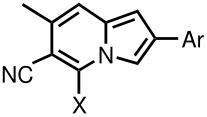				
No.	5-X*	R in 2-Ar	Yield %	m.p., °C

**2 a**	Cl	p-F	30	173–175
**2 b**	Cl	p-Cl	65	198–200
**2 c**	Cl	p-Br	54	229–230
**2 d**	Cl	p-Me	74	157–158
**3 a**	OMe	p-Cl	50	169–172
**3 b**	OMe	p-Br	71	197–200
**3 c**	OMe	p-Me	73	170–173
**4 a**	pyrrolidyl	p-F	99	189–190
**4 b**	piperidyl	p-F	91	205–209
**4 c**	hexamethy-lenimino	p-F	88	186–188
**4 d**	benzylamino	p-F	58	190–194
**4 e**	pyrrolidyl	p-Me	74	228–230
**4 f**	piperidyl	p-Me	71	212–215
**4 g**	hexamethy-lenimino	p-Me	85	213–216
**4 h**	benzylamino	p-Me	83	185–187
**5 a**	S(CH_2_)_2_OH	p-Me	71	132–135
**5 b**	SCH_2_CO_2_Et	p-F	70	142–145

## Note

*Characteristics of parent indolizinones **1 a** – **d** were identical to those described in literature.[9]

^†^ Supporting information

## Supporting Information

File 1 Supporting tables

File 2 Supporting information
