# Supplementary material for: Efficient synthesis of 5-substituted 2-aryl-6-cyanoindolizines via nucleophilic substitution reactions
Source: Beilstein J Org Chem. 2005 Oct 7;1:9. doi: 10.1186/1860-5397-1-9 (PMC1399457; doi:10.1186/1860-5397-1-9)
Supplement: File 1 — Supporting tables [file Beilstein_J_Org_Chem-01-09-s001.doc]

Table 2a. ^1^H NMR chemical shifts in 2-aryl-6-cyano-7-methyl-5-indolizinones **1 a** - **d**, 2-aryl-5-chloro-6-cyano-7-methylindolizines **2 a** - **d**, and 6-cyano-2-(*p*-fluoro)-7-methyl-5-indolizinthione **6** (δ; DMSO)

| No. | Ar | H_1_ | H_3_ | H_8_ | C_6_H_4_ | 7-Me | p-Me |
| --- | --- | --- | --- | --- | --- | --- | --- |
| **1 a** | *p*-F-C_6_H_4_ | 7.28 (1H) | 5.19 (2H) | 6.54 (1H) | m., 7.86 (2H); m., 7.28 (2H) | 2.43 (3H) |  |
| **1 b** | *p*-Cl-C_6_H_4_ | 7.35 (1H) | 5.19 (2H) | 6.55 (1H) | m., 7.82 (2H); m., 7.51 (2H) | 2.44 (3H) |  |
| **1 c** | *p*-Br-C_6_H_4_ | 7.29 (1H) | 5.60 (2H) | 6.49 (1H) | m., 8.02 (2H); m., 7.61 (2H) | 2.44 (3H) |  |
| **1 d** | *p*-Me- C_6_H_4_ | 7.20 (1H) | 5.15 (2H) | 6.49 (1H) | m., 7.67 (2H); m., 7.29 (2H) | 2.46 (3H) | 2.40 (3H) |
| **2 a** | *p*-F-C_6_H_4_ | 7.42 (1H) | 8.06 (1H) | 6.95 (1H) | m., 7.81 (2H); m., 7.18 (2H) | 2.44 (3H) |  |
| **2 b** | *p*-Cl-C_6_H_4_ | 7.42 (1H) | 8.11 (1H) | 6.98 (1H) | m., 7.79 (2H); m., 7.41 (2H) | 2.44 (3H) |  |
| **2 c** | *p*-Br-C_6_H_4_ | 7.40 (1H) | 8.19 (1H) | 6.96 (1H) | m., 7.72 (2H); m., 7.56 (2H) | 2.45 (3H) |  |
| **2 d** | *p*-Me- C_6_H_4_ | 7.39 (1H) | 7.99 (1H) | 6.92 (1H) | m., 7.63 (2H); m., 7.21 (2H) | 2.44 (3H) | 2.37 (3H) |
| **6** | *p*-F-C_6_H_4_ | 7.44 (1H) | 5.35 (2H) | 7.05 (1H) | m., 7.90 (2H); m., 7.27 (2H) | 2.54 (3H) |  |

Table 2b. ^1^H NMR chemical shifts and mass-spectral data for 5-substituted 2-aryl-6-cyano-7-methylindolizines

| No. | 5-X | R in Ar | M^+^ | ^1^H-NMR chemical shifts (δ, DMSO) |
| --- | --- | --- | --- | --- |
| **2 a** | Cl | p-F | 284 | See Table 1 |
| **2 b** | Cl | p-Cl | 300 | See Table 1 |
| **2 c** | Cl | p-Br |  | See Table 1 |
| **2 d** | Cl | p-Me | 280 | See Table 1 |
| **3 a** | OMe | p-Cl | 296 | 7.93 (1H); 7.73 (2H); 7.38 (2H); 7.04 (1H); 6.74 (1H); 4.42 (3H); 2.37 (3H) |
| **3 b** | OMe | p-Br | 340 | 7.94 (1H); 7.68 (2H); 7.53 (2H); 7.06 (1H); 6.77 (1H); 4.44 (3H); 2.38 (3H) |
| **3 c** | OMe | p-Me | 276 | 7.82 (1H); 7.59 (2H); 7.19 (2H); 7.04 (1H); 6.71 (1H); 4.43 (3H); 2.37 (3H); 2.36 (3H) |
| **4 a** | pyrrolidyl | p-F | 319 | 7.77 (3H); 7.15 (2H); 7.05 (1H); 6.68 (1H); 3.67 (4H); 2.36 (3H); 2.15 (4H) |
| **4 b** | piperidyl | p-F | 333 | 7.72 (2H); 7.61 (1H); 7.14 (2H); 7.08 (1H); 6.70 (1H); 3.40 (4H); 2.36 (3H); 1.87 (6H) |
| **4 c** | hexamethylenimino | p-F | 347 | 7.72 (3H); 7.14 (2H); 6.72 (1H); 3.49 (4H); 2.35 (3H); 1.88 (8H) |
| **4 d** | benzylamino | p-F | 355 | 8.17 (1H); 7.24-7.71 (7H); 7.15 (2H); 6.68 (1H); 6.54 (1H); 5.05 (2H); 2.26 (3H) |
| **4 e** | pyrrolidyl | p-Me | 315 | 7.70 (1H); 7.59 (2H); 7.18 (2H); 7.03 (1H); 6.65 (1H); 3.65 (4H); 2.36 (3H); 2.35 (3H); 2.14 (4H) |
| **4 f** | piperidyl | p-Me | 329 | 7.58 (1H); 7.56 (2H): 7.18 (2H); 7.05 (1H); 6.68 (1H); 3.39 (4H); 2.36 (3H); 2.34 (3H); 1.86 (6H) |
| **4 g** | hexamethylenimino | p-Me | 343 | 7.70 (1H); 7.58 (2H); 7.19 (2H); 7.09 (1H); 6.70 (1H); 3.48 (4H): 2.38 (3H); 2.36 (3H); 1.88 (8H) |
| **4 h** | benzylamino | p-Me | 351 | 8.17 (1H); 7.25-7.63 (7H); 7.19 (2H); 6.67 (1H); 6.54 (1H); 5.04 (2H); 2.36 (3H); 2.27 (3H) |
| **5 a** | S(CH_2_)_2_OH | p-Me | 322 | 8.21 (1H); 7.63 (2H); 7.38 (1H); 7.21 (2H); 6.86 (1H); 4.82 (1H); 3.66 (2H); 3.21 (2H); 2.41 (3H); 2.37 (3H) |
| **5 b** | SCH_2_CO_2_Et | p-F | 368 | 8.20 (1H); 7.80 (2H); 7.45 (1H); 7.18 (2H); 6.90 (1H); 4.04 (2H); 3.90 (2H); 2.43 (3H) 1.12 (3H) |
