# Supplementary figures and images for: Efficient synthesis of 5-substituted 2-aryl-6-cyanoindolizines via nucleophilic substitution reactions
Source: Beilstein J Org Chem. 2005 Oct 7;1:9. doi: 10.1186/1860-5397-1-9 (PMC1399457; doi:10.1186/1860-5397-1-9)

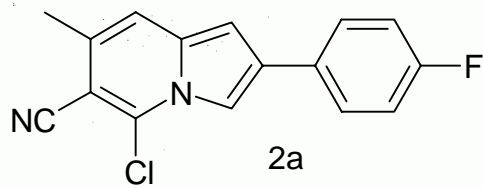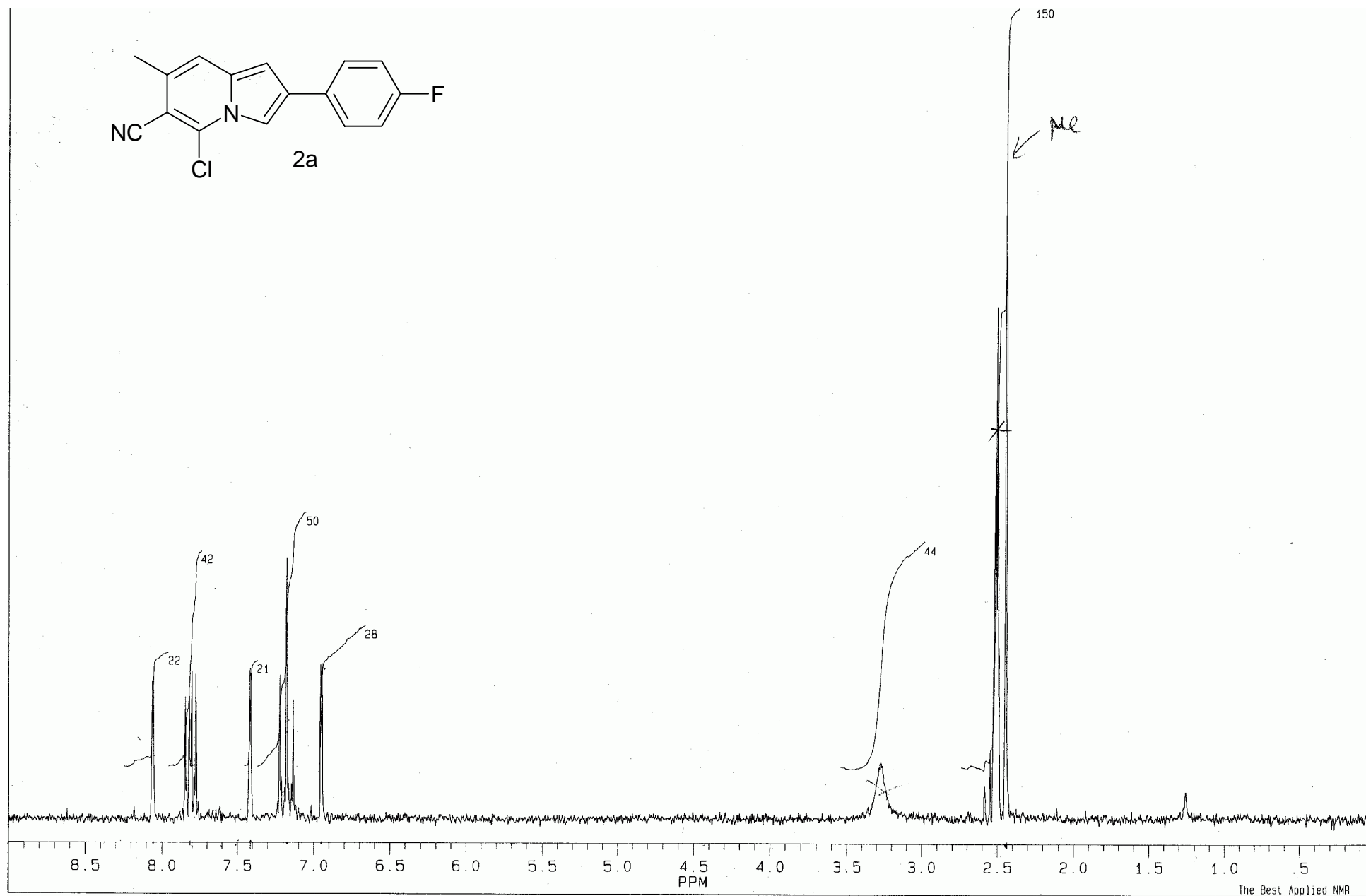

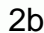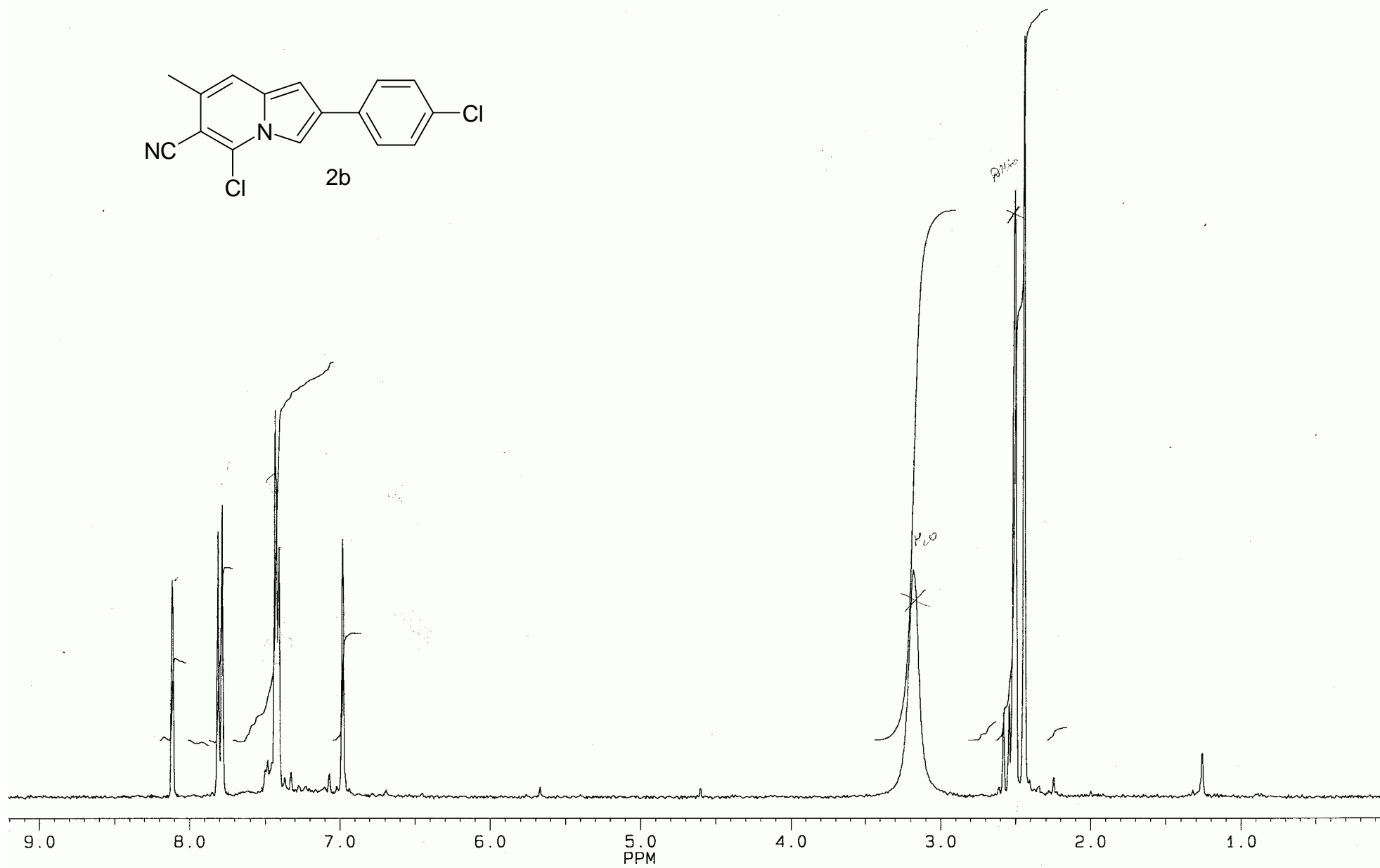

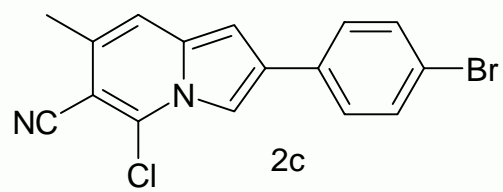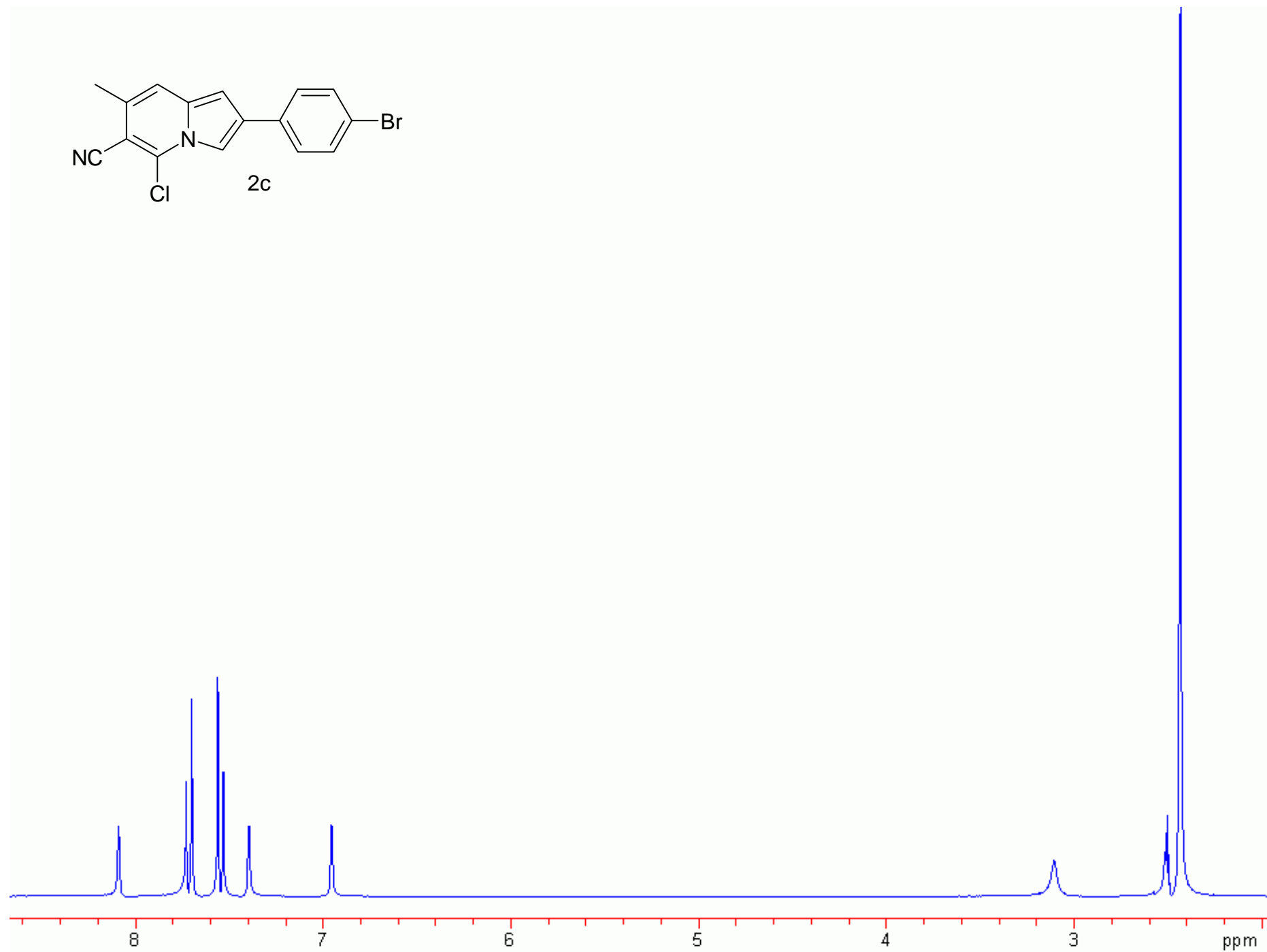

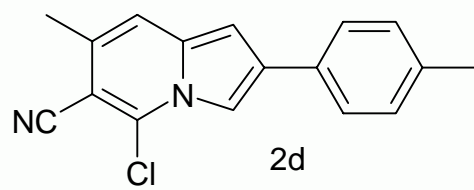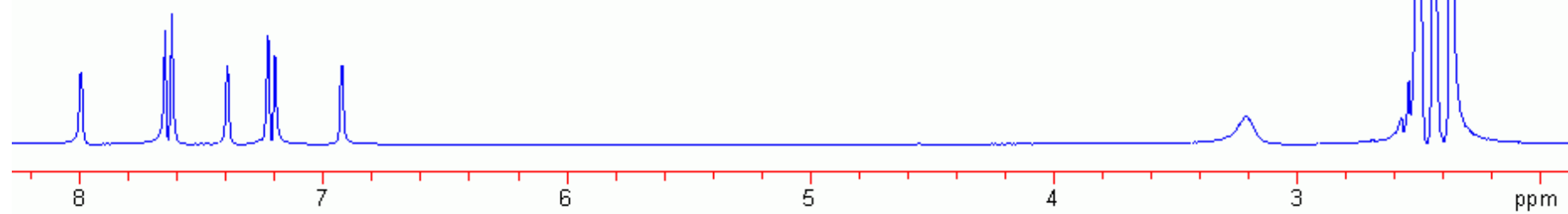

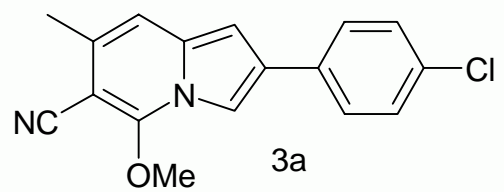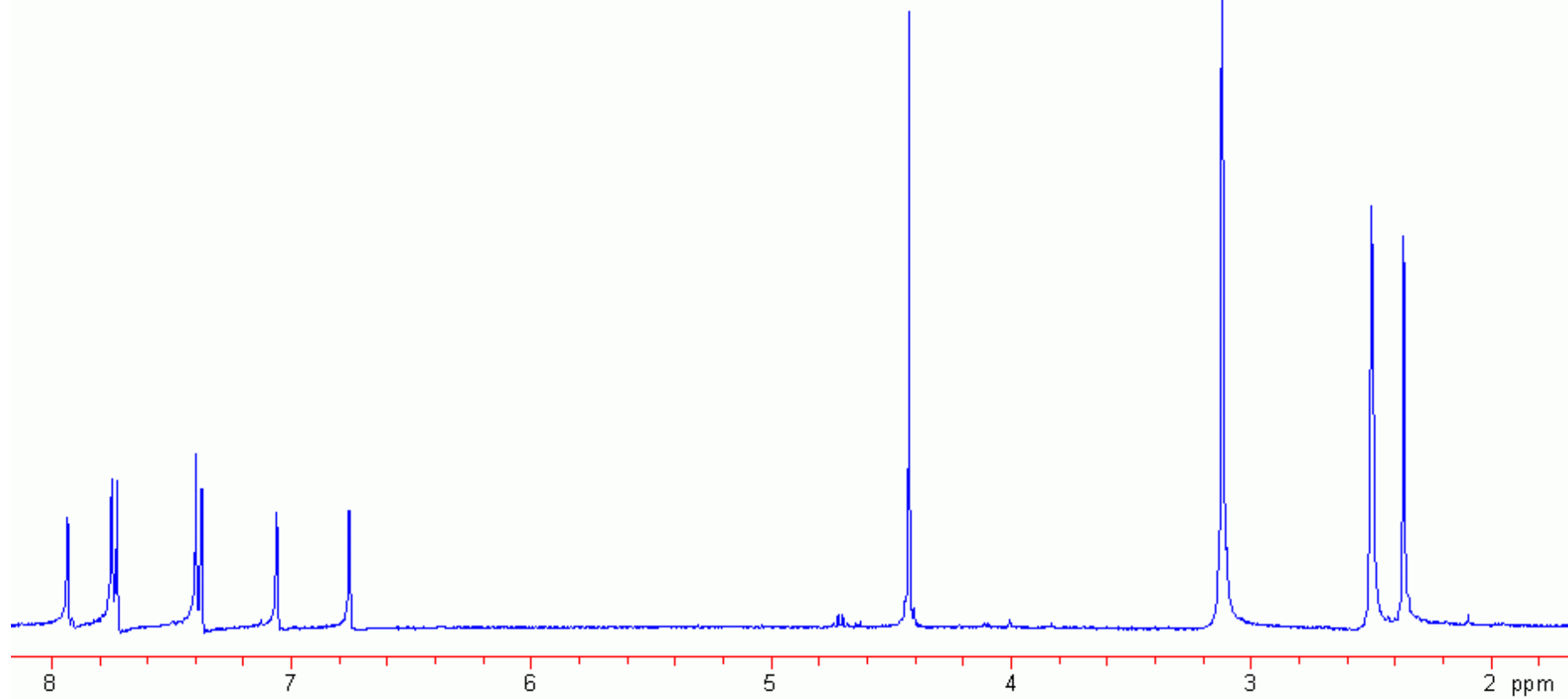

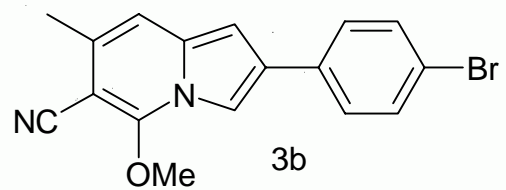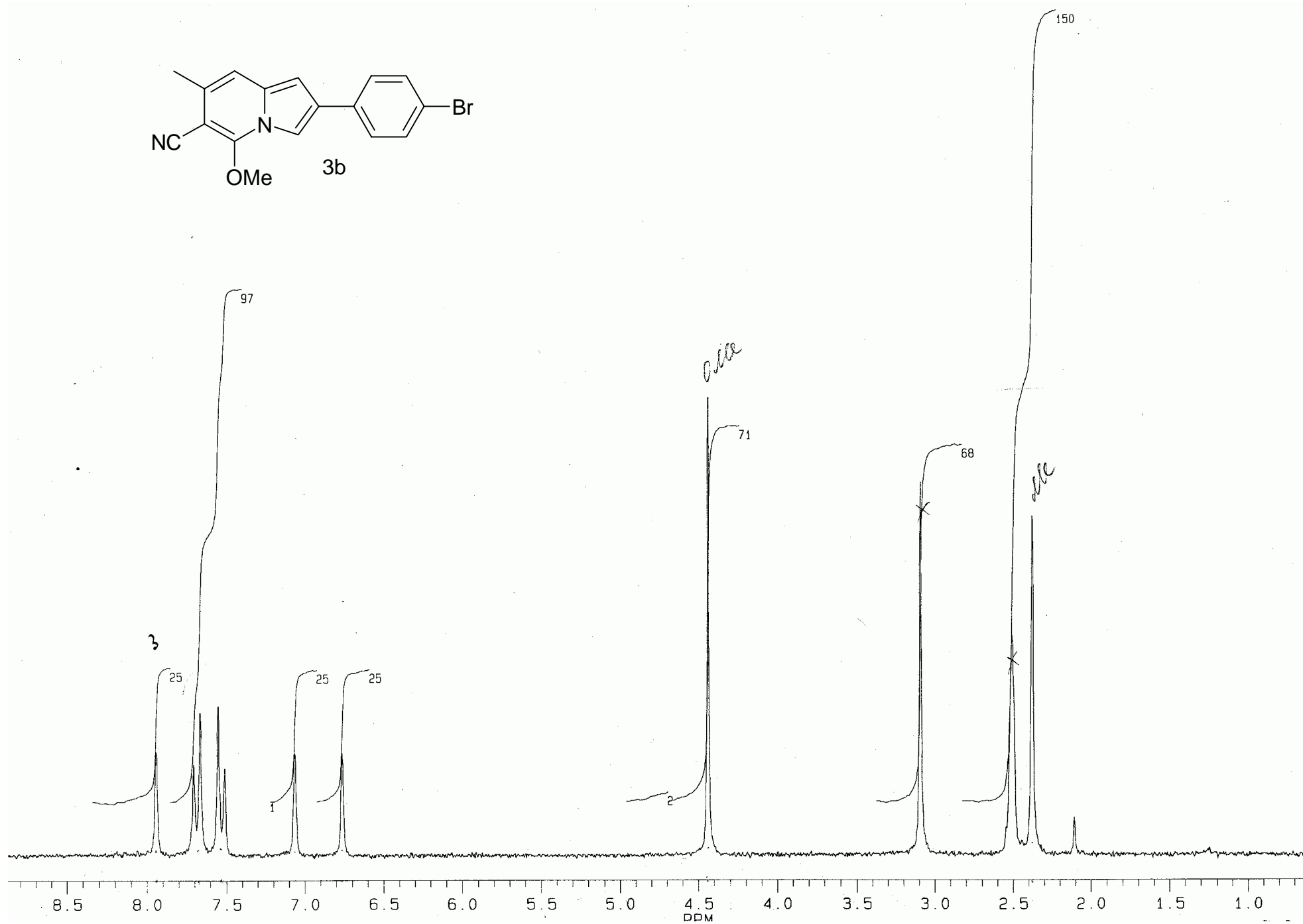

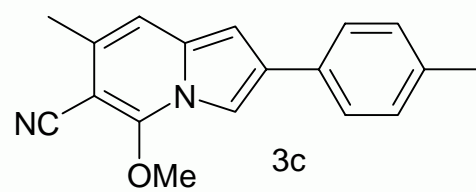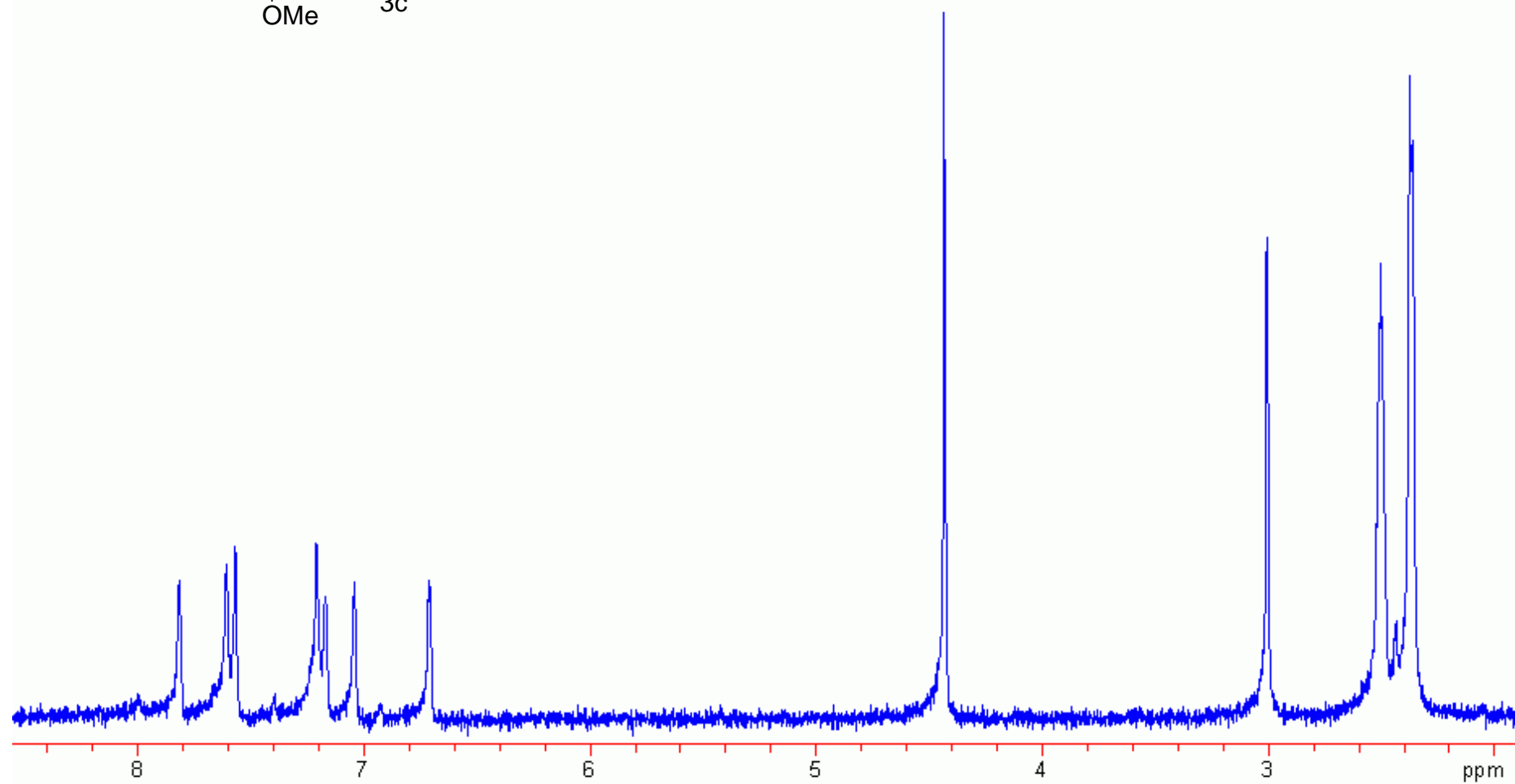

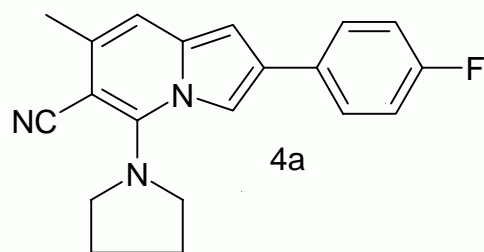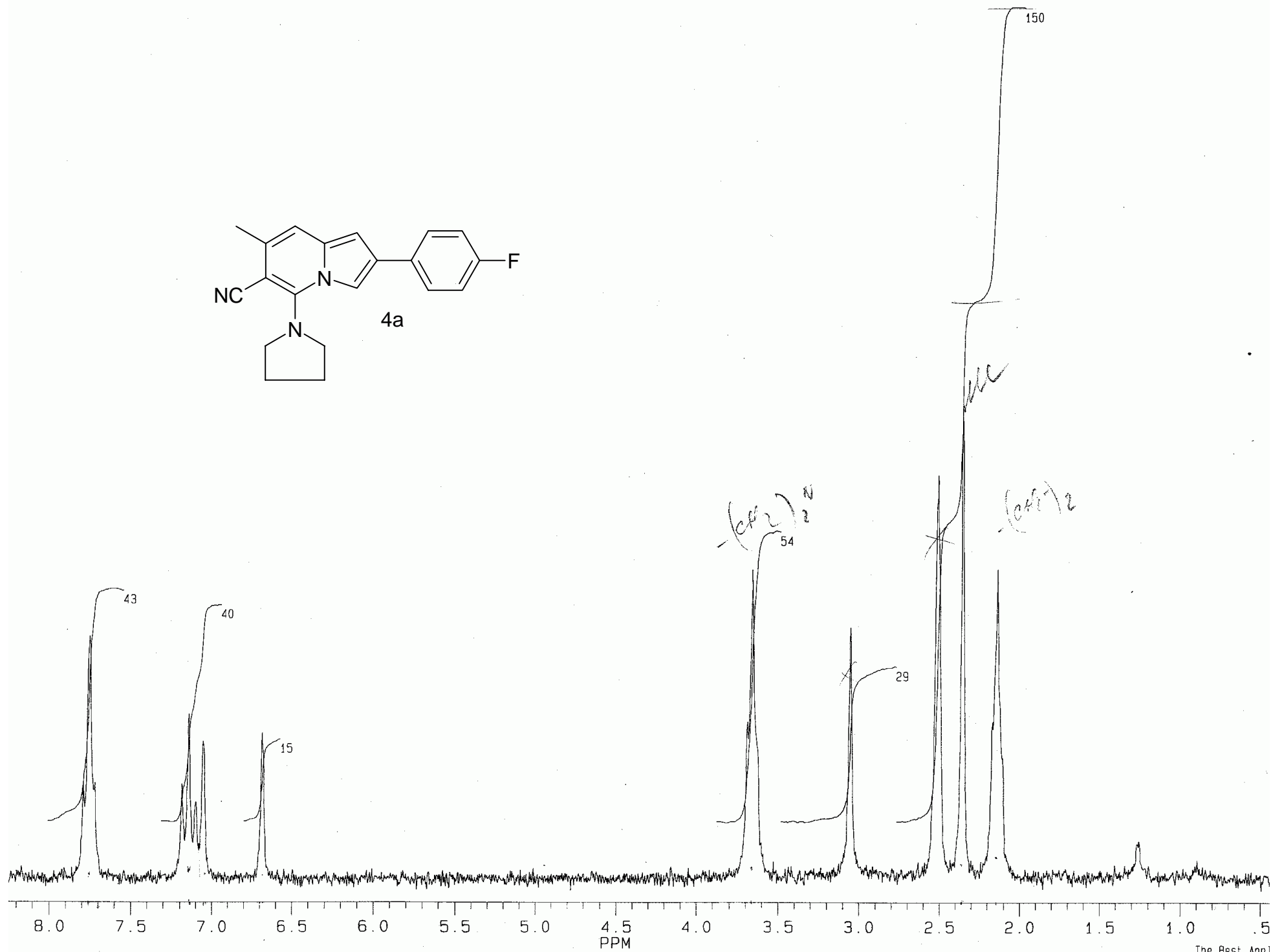

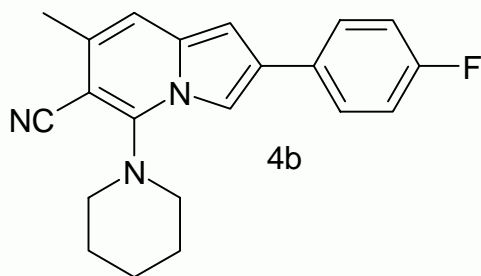

4b

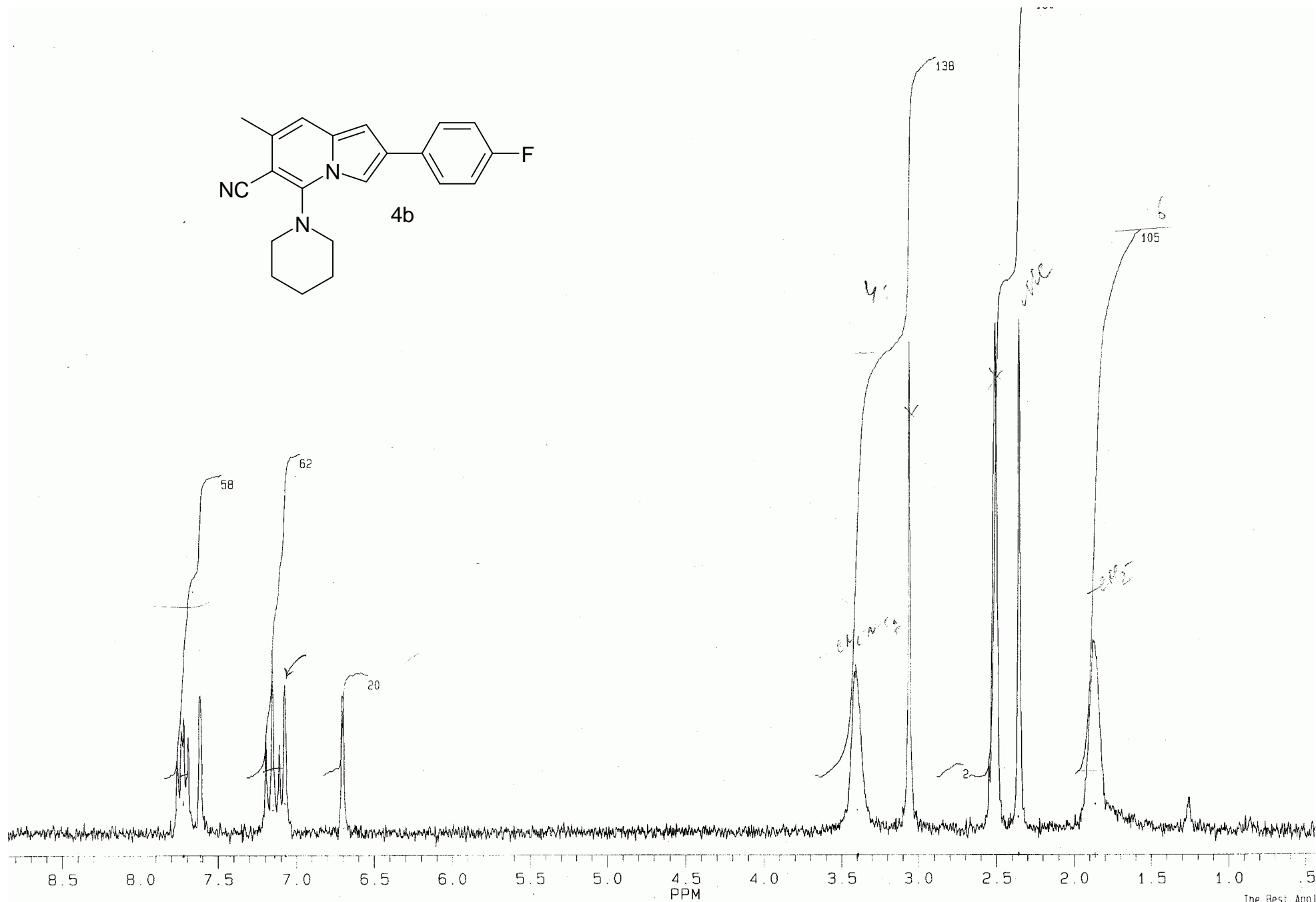

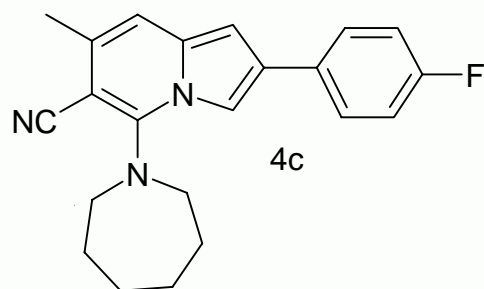

4c

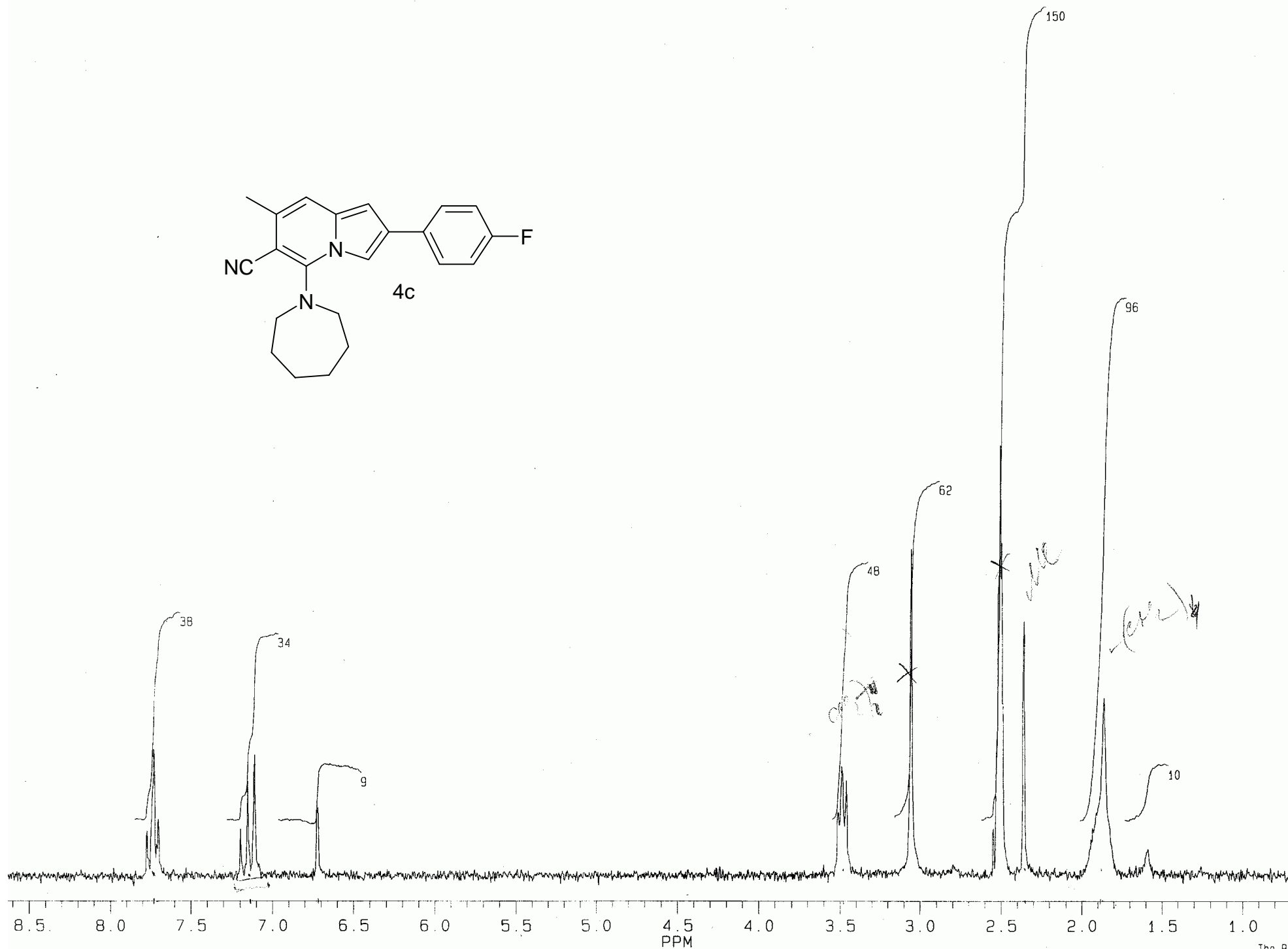

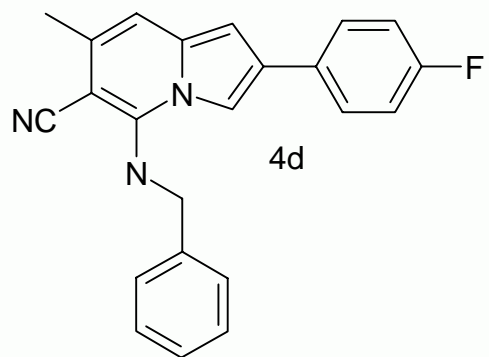

4d

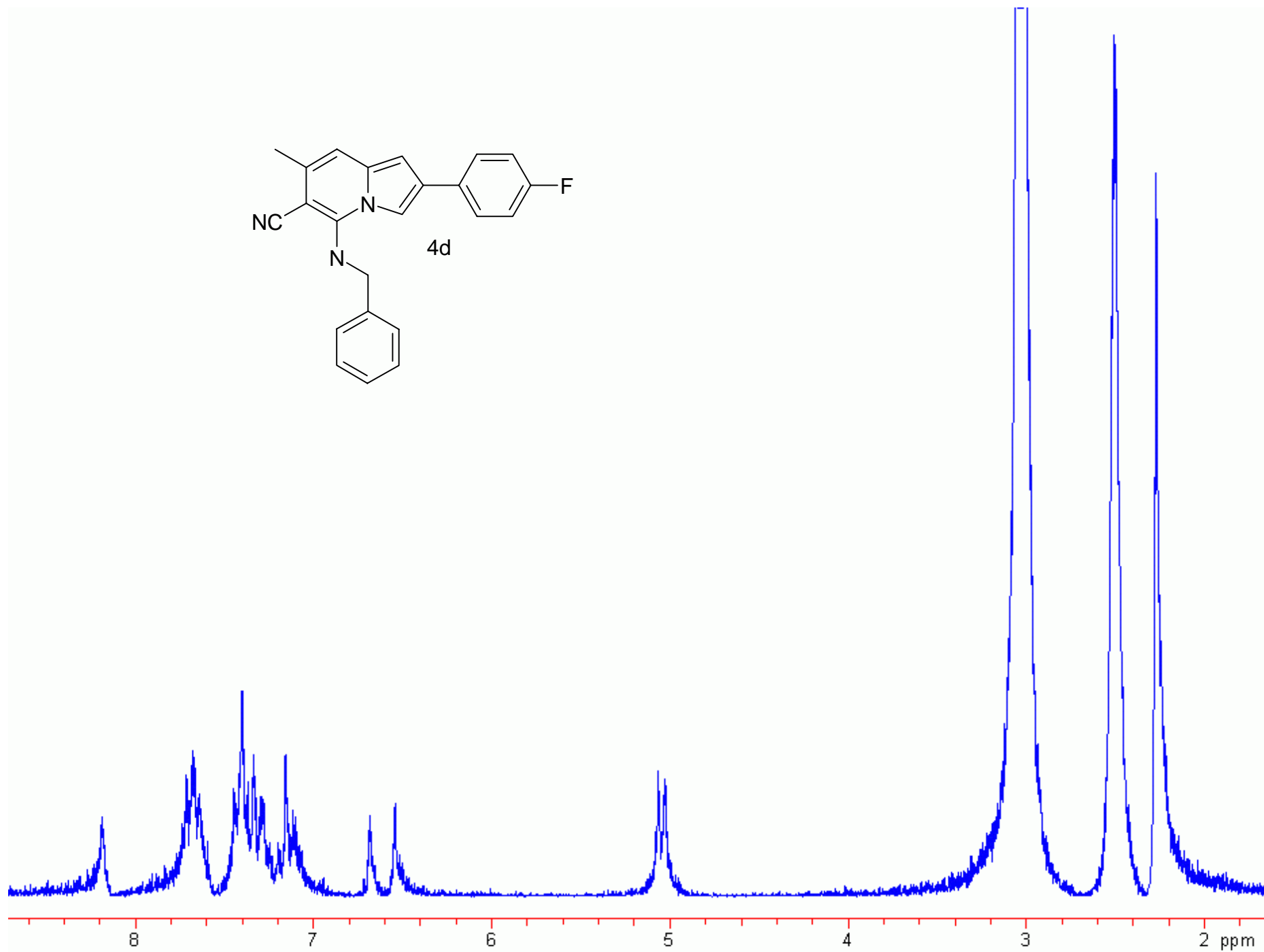

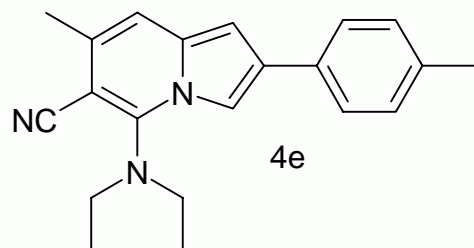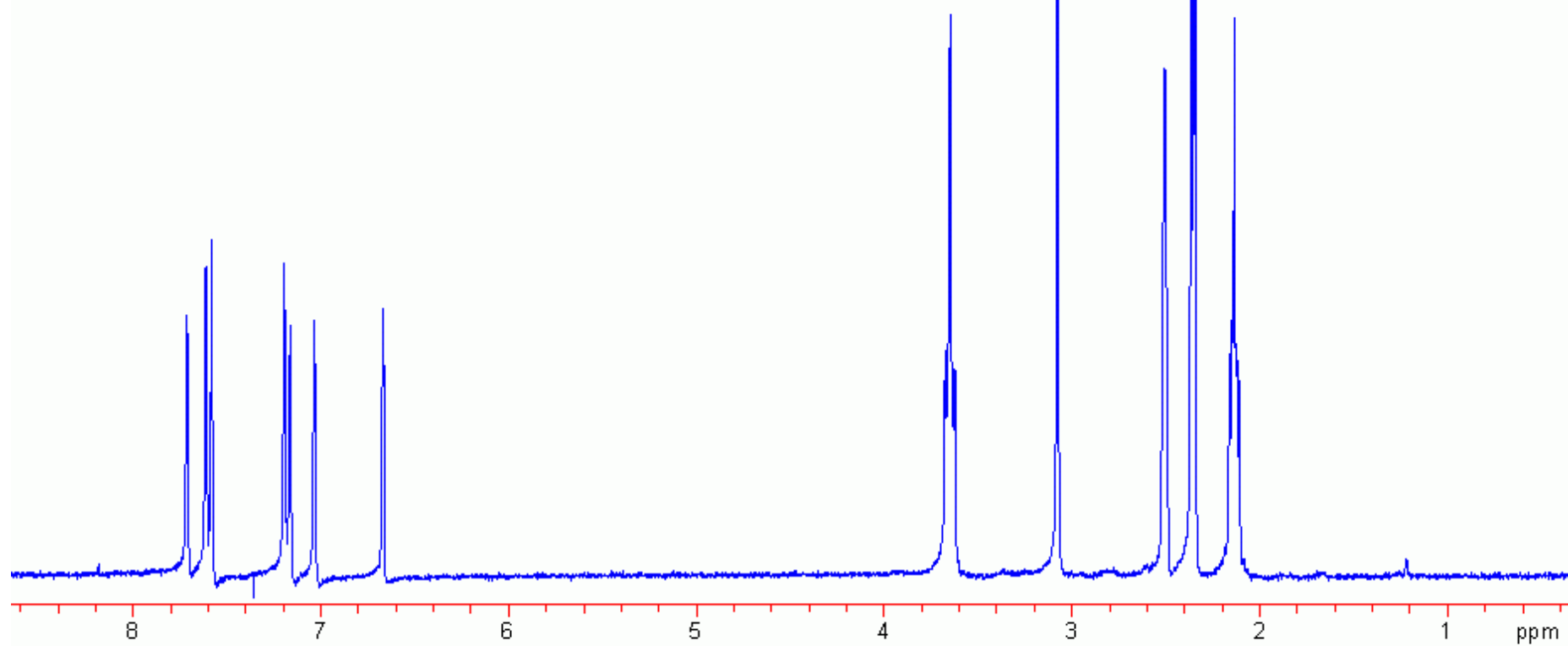

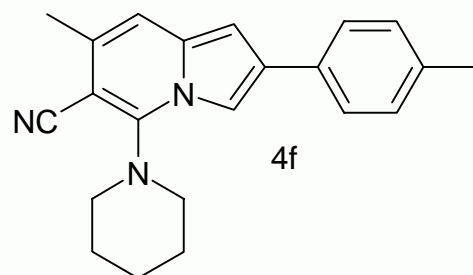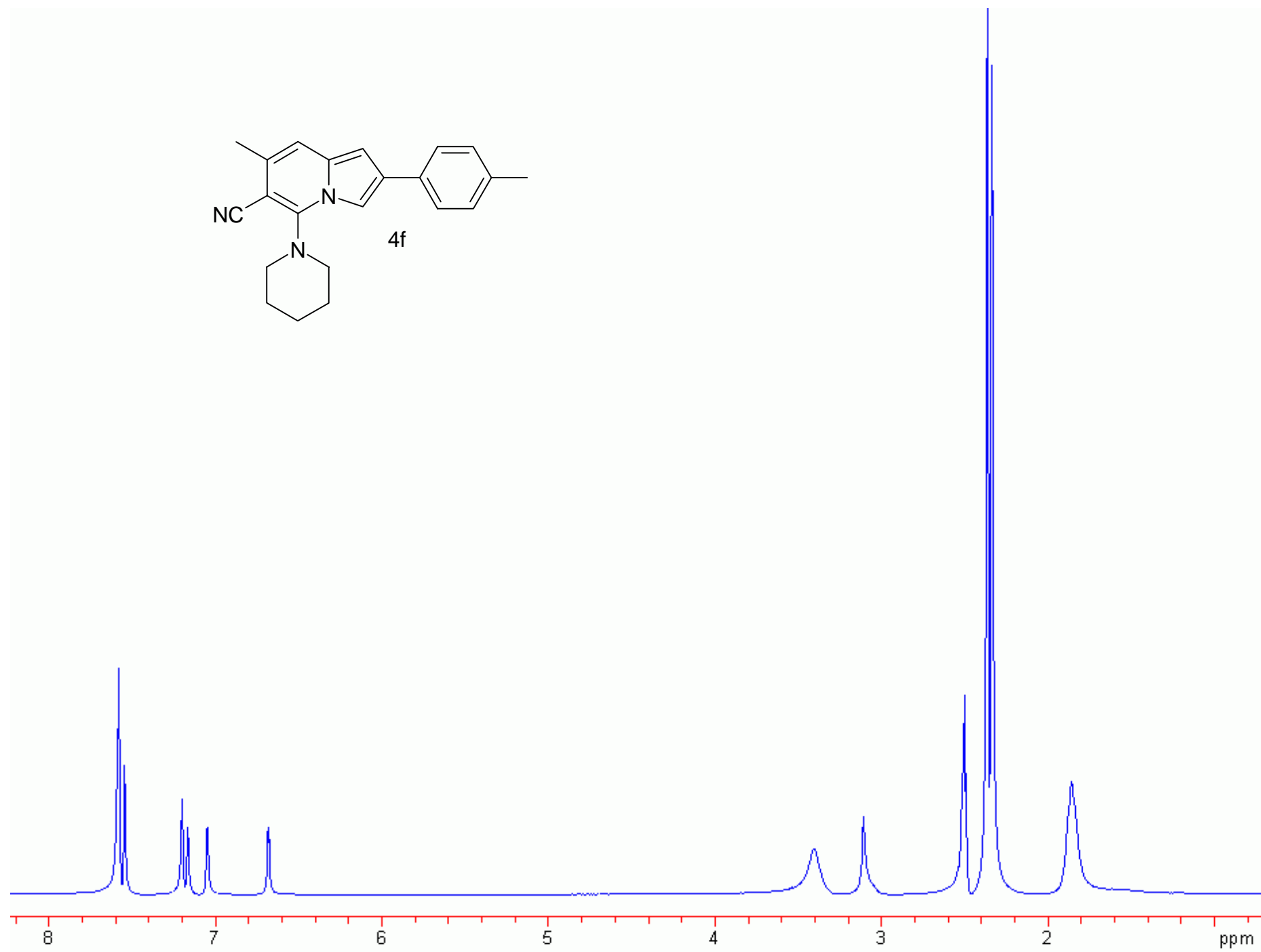

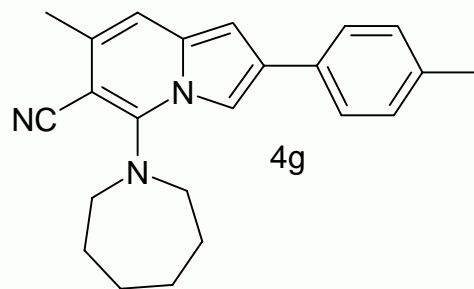

4g

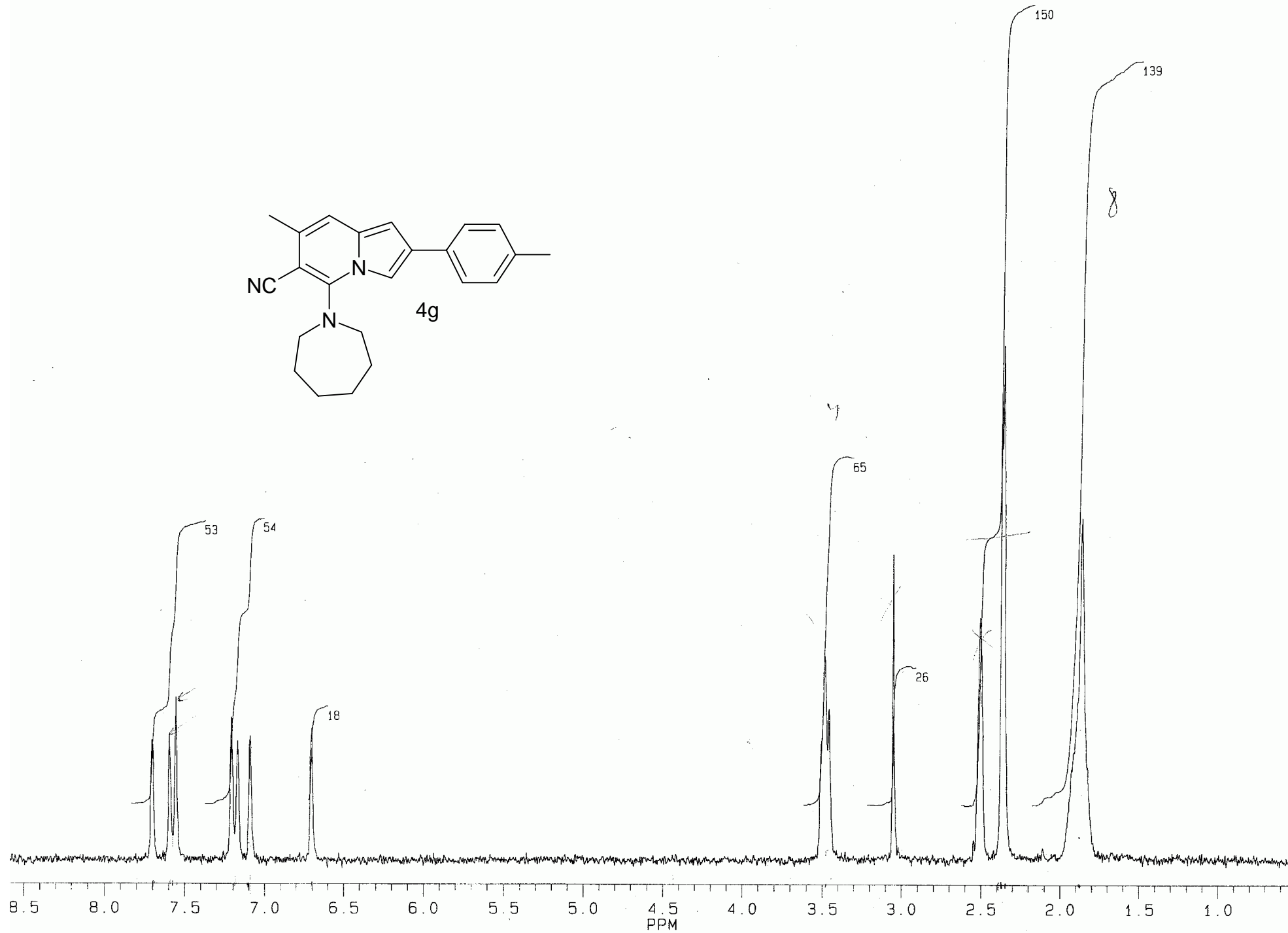

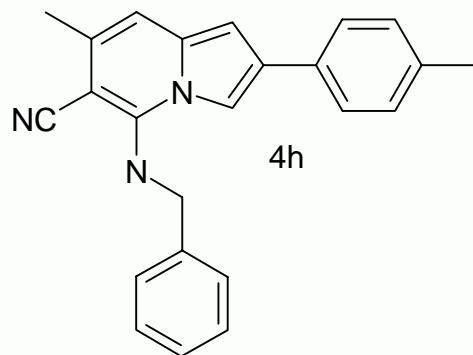

4h

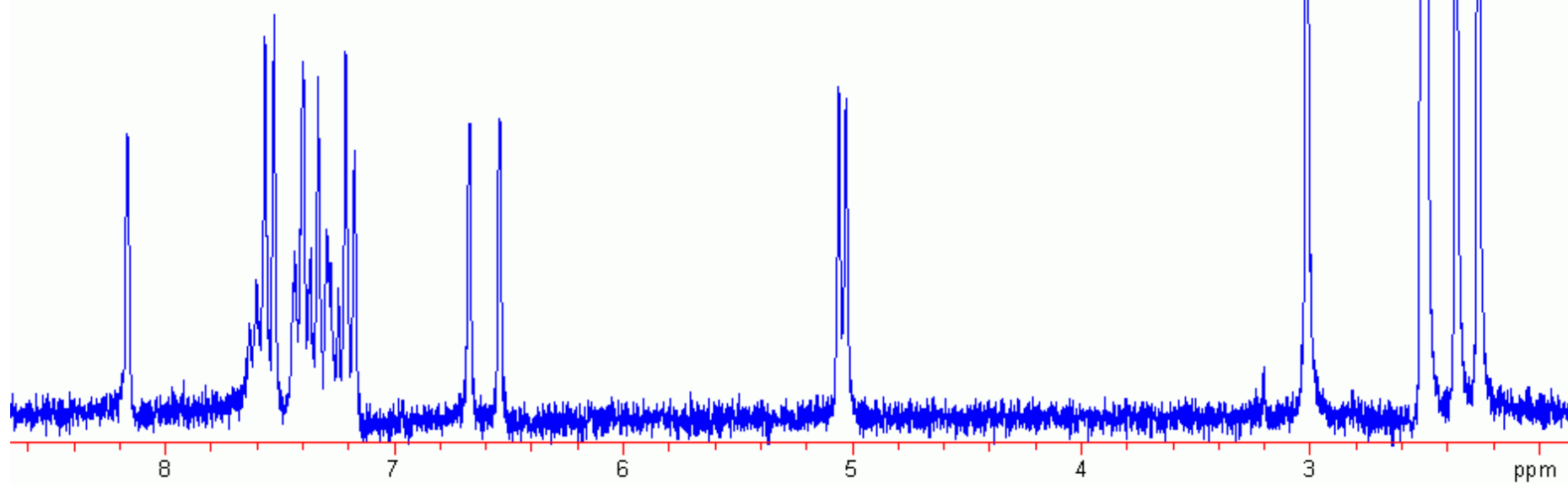

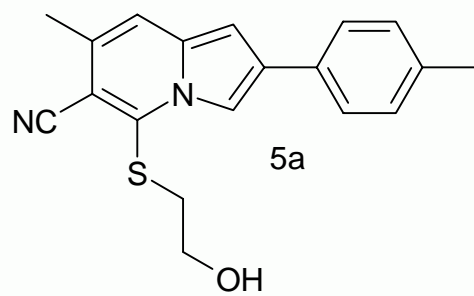

5a

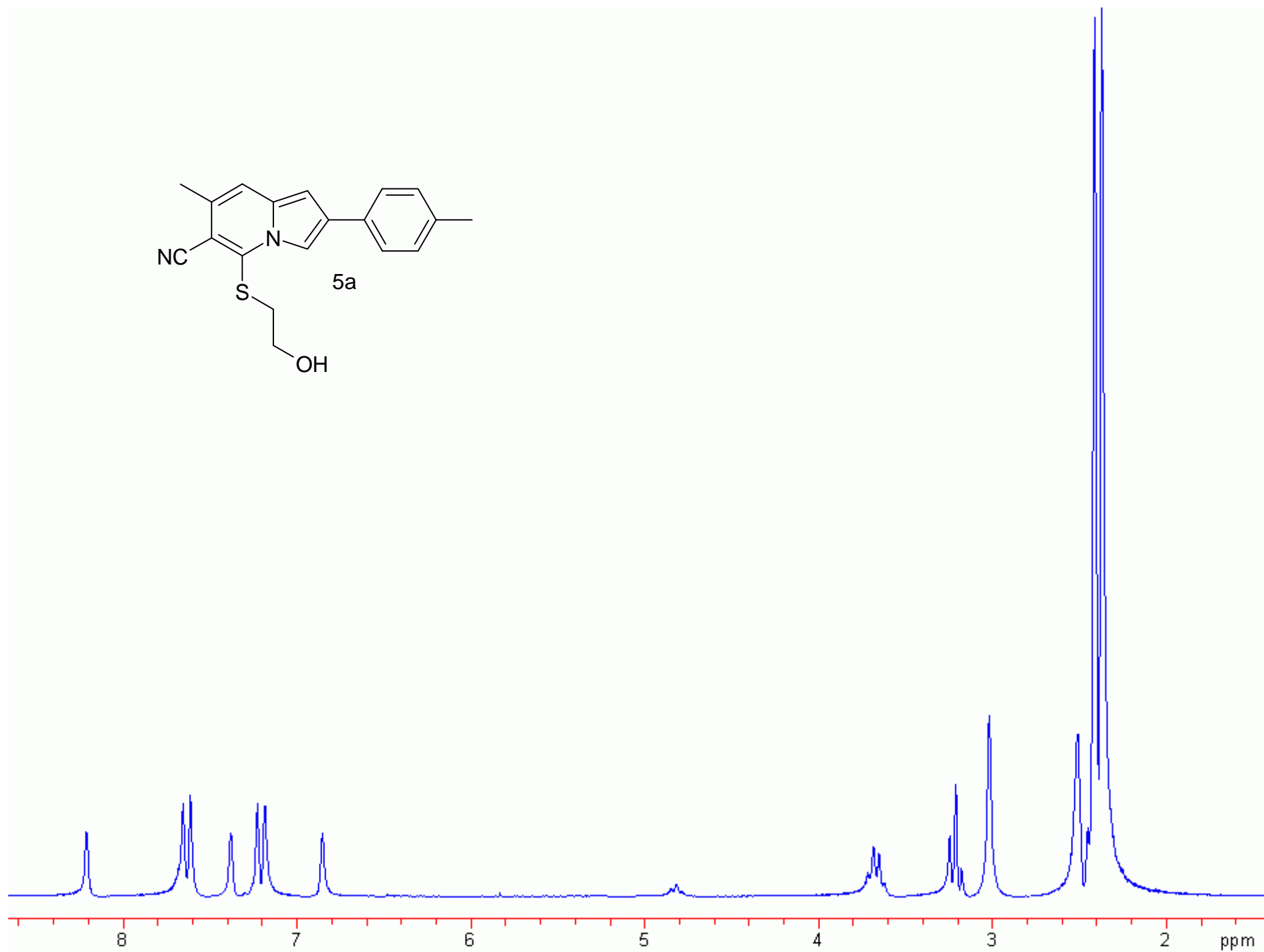

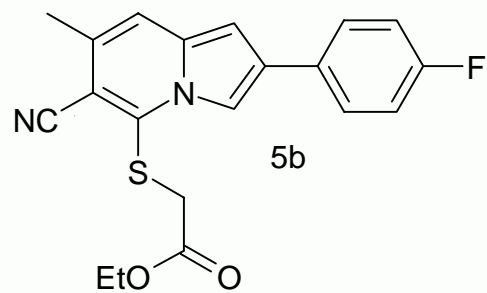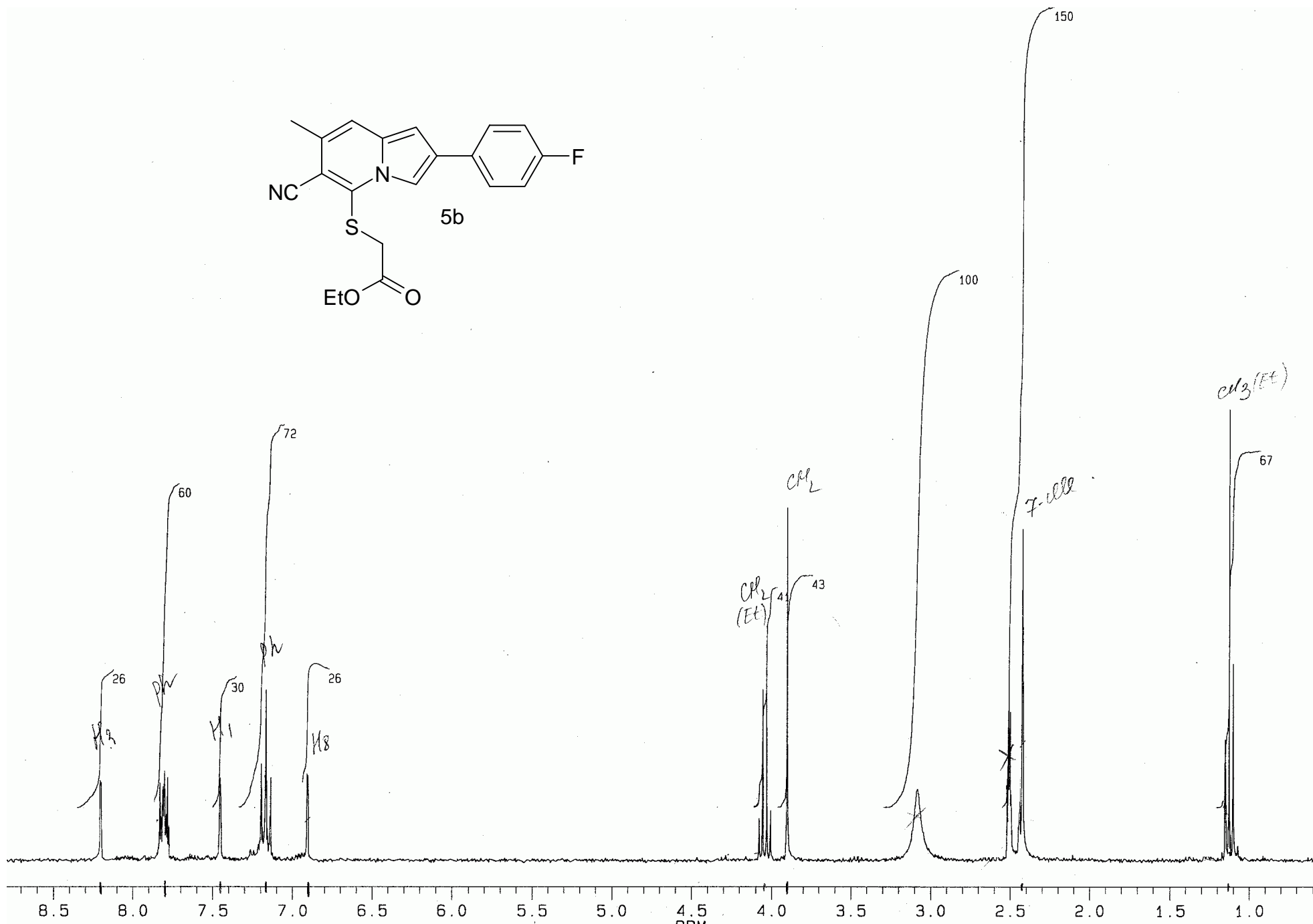

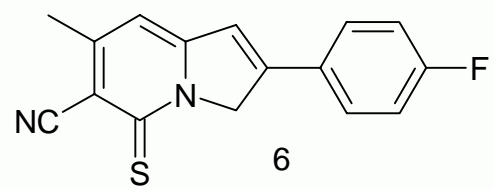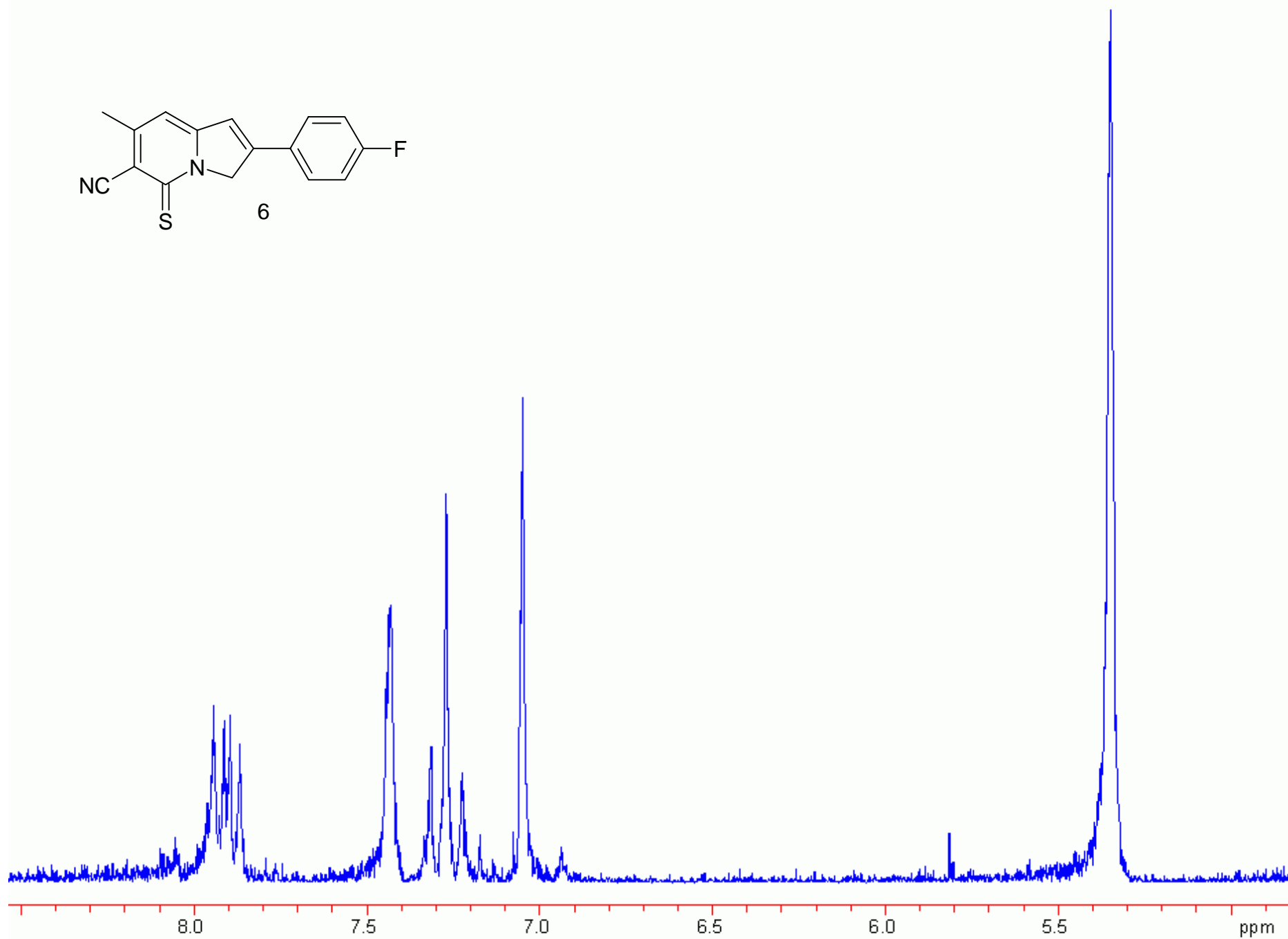

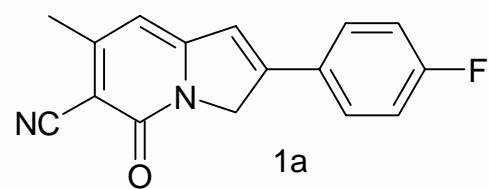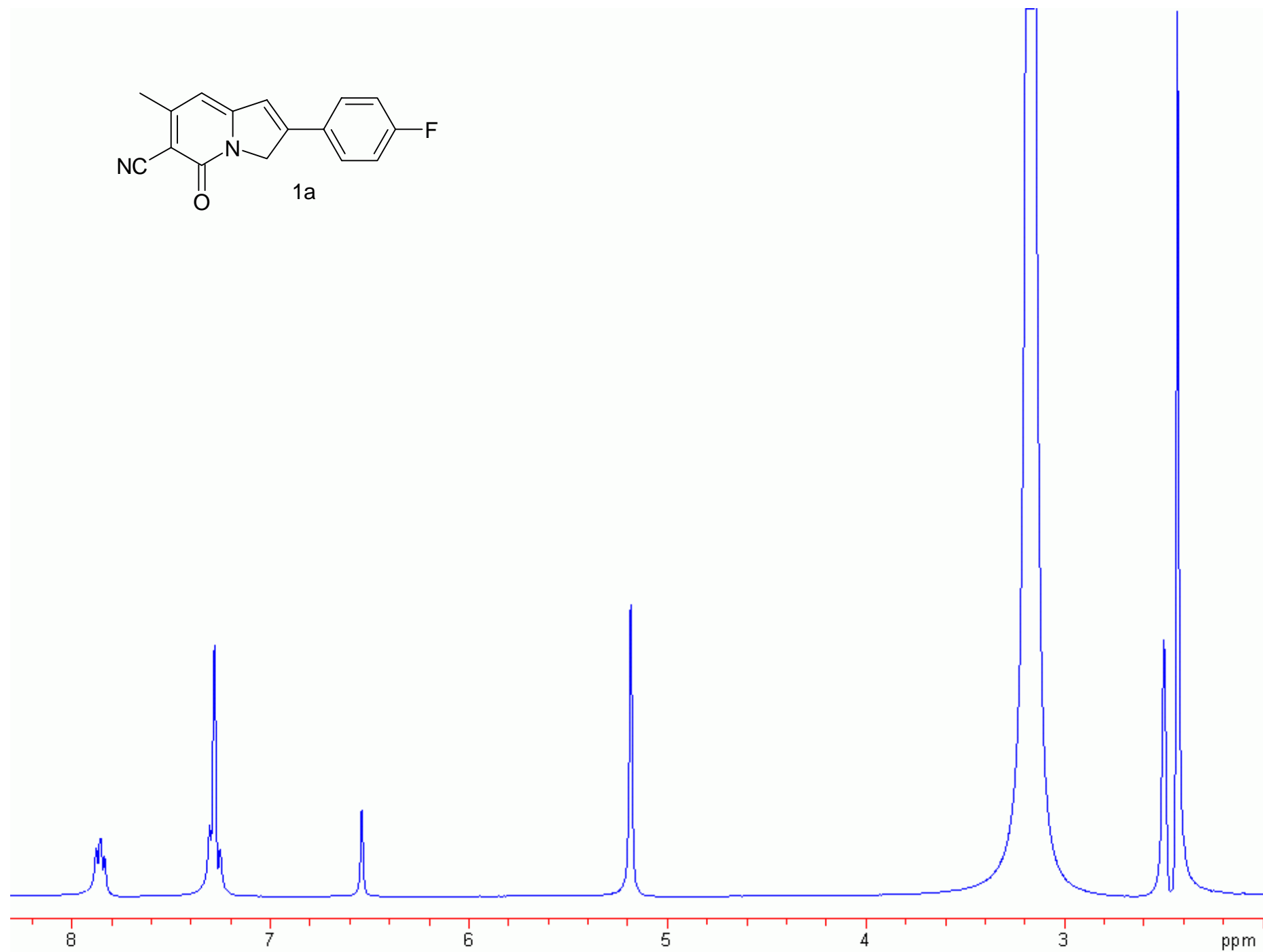

Supplement: File 2 — Supporting information [file Beilstein_J_Org_Chem-01-09-s002.pdf]
